# Effects of Maternal Vitamin D Levels on Prematurity: Feasibility Study in a Multicenter Observational Pilot

**DOI:** 10.3390/nu17071160

**Published:** 2025-03-27

**Authors:** Olivia Barbosa, Ana Teresa Freitas, Marta P. Silvestre, André Moreira-Rosário, Pedro Aguiar, Ana Isabel Régua, Tatiana Madaleno, Manuela Almeida, Dulce Cruz

**Affiliations:** 1 Comprehensive Health Research Centre (CHRC), 1150-082 Lisboa, Portugal; dcruz@uevora.pt; 2 Escola Superior de Enfermagem São João de Deus, Universidade de Évora, 7000-811 Évora, Portugal; 3 Neonatal Intensive Care Unit, Unidade Local de Saúde do Alentejo Central (ULSAC), 7000-811 Évora, Portugal; 4 Instituto de Engenharia de Sistemas e Computadores, Investigação e Desenvolvimento em Lisboa (INESC-ID), 1000-029 Lisboa, Portugal; ana.freitas@tecnico.ulisboa.pt; 5 Instituto Superior Técnico, Universidade de Lisboa, 1049-001 Lisboa, Portugal; 6 NOVA Medical School, Universidade NOVA de Lisboa, 1169-056 Lisboa, Portugal; marta.silvestre@nms.unl.pt (M.P.S.); andre.rosario@nms.unl.pt (A.M.-R.); 7 Centro de Investigação em Tecnologias e Serviços de Saúde (CINTESIS), NOVA Medical School, Universidade NOVA de Lisboa, 1169-056 Lisboa, Portugal; 8 NOVA National School of Public Health, Universidade NOVA de Lisboa, 1600-560 Lisboa, Portugal; pedroaguiar@ensp.unl.pt; 9 Unidade Local de Saúde do Baixo Alentejo (ULSBA), 7801-849 Beja, Portugal; ana.regua@ulsba.min-saude.pt; 10 Unidade Local de Saúde do Norte Alentejo (ULSNA), 7301-853 Portalegre, Portugal; tatiana.madaleno@ulsaale.min-saude.pt; 11 Outpatient Consultation Service, Unidade Local de Saúde do Alentejo Central (ULSAC), 7005-169 Évora, Portugal; mcouquinha@hevora.min-saude.pt

**Keywords:** feasibility study, maternal vitamin D levels, prematurity, public health

## Abstract

**Background:** Numerous studies have shown that hypovitaminosis D is linked to adverse maternal and infant health outcomes, such as intrauterine growth restriction, preeclampsia, cholestasis, hypertension, and gestational diabetes, all of which are potential causes of prematurity. Recognizing the significance of this issue and its impact on maternal–infant health, the VitDTracking research project was designed and registered on 4 March 2024, in the ClinicalTrials.gov database (Identifier: NCT06292195). The project includes a large-scale multicenter observational study, targeting a minimum initial sample size of 1800 participants. This paper describes the pilot study aimed at assessing the feasibility of the full-scale study. **Methods**: A multicenter observational pilot study was conducted in public health organizations in the Alentejo region, adhering to the research protocol. Data collection included 67 parameters: 37 obtained from a questionnaire and 30 from clinical records, with particular focus on maternal 25(OH)D levels and maternal–infant health outcomes. Feasibility was assessed through predefined outcome indicators and success criteria. **Results**: The pilot study enrolled 30 pregnant women and successfully met all feasibility criteria. The global recruitment rate was 73.17%, with an eligible recruitment rate of 88.2%. The refusal rate was low (11%), and adherence, retention, and completion rates were all 100%, indicating strong participant engagement. The questionnaire comprehension rate was 86.6%. Participating centers demonstrated the capacity to implement the study, adhering to the protocol with a compliance rate exceeding 90%. The study also highlighted a concerning prevalence of hypovitaminosis D and identified cases of premature birth and miscarriage. **Conclusions**: The pilot study confirmed the feasibility of recruitment methodologies and procedures, supporting the implementation of the large-scale observational study. The planned study will recruit approximately 1800 pregnant women to achieve an eligible cohort of 1000 samples, and a statistically significant final sample of 100 cases meeting the prematurity criterion.

## 1. Introduction

The prevalence of hypovitaminosis D in pregnant women is significantly high all over the world, with the highest prevalence rates (>80%) reported in countries with high sun exposure, such as China, Turkey, Iran, and Pakistan [1,2]. The prevalence remains high even with a supplementation dose of 400 to 600 IU/day during pregnancy (the dose usually recommended by obstetricians in Portugal), which is considered as a suboptimal dose [3,4]. In 2020, an observational study was conducted in Switzerland to assess serum 25-hydroxyvitamin D [25(OH)D] levels in pregnant women during their first trimester who were taking the recommended supplementation of 600 IU/day of vitamin D. The study found that, out of the 1382 pregnant women included in the research, 73.23% had vitamin D deficiency, with serum 25(OH)D levels below 20 ng/mL [5].

Several studies show that vitamin D deficiency is associated with adverse maternal and infant outcomes, such as intrauterine growth restriction (IUGR), preeclampsia, cholestasis, hypertension and gestational diabetes, potential causes of prematurity [5,6,7,8].

An adequate maternal level of 25(OH) D is essential for good health outcomes on the mother and on the fetus, with a potential ability to modulate maternal and fetal gene expression. Fetal and neonatal vitamin D levels are dependent on maternal levels and their interaction with epigenetic mechanisms in early fetal life, which may help explain their non-classical benefits, including a lower risk of infants being small for gestational age, improved growth during infancy, enhanced immune function, and reduced susceptibility to chronic diseases later in life [9,10,11]. The 25(OH)D easily crosses the placenta and is activated into 1,25-dihydroxyvitamin D [1,25(OH)^2^ D] by fetal kidneys. If maternal levels are sufficient, 1,25(OH)^2^ D can also be synthesized inside the placenta to regulate placental metabolism through the vitamin D receptor (VDR) constituting an important source of this micronutrient. Vitamin D deficiency can impair placental formation and development, negatively impacting maternal–fetal metabolism, which directly influences gestational duration and birth weight [10,12].

Vitamin D deficiency and insufficiency can have many causes, but the genetic component plays a highly significant role [13,14]. Nutrition, as a determinant of health, is considered to be the main environmental factor involved in modulating gene expression, right from the intrauterine stage. Among nutrients, the importance of vitamin D stands out as one of the epigenetic determinants that most influences the health-disease process, from fetal development onwards [15,16]. The biological effects of vitamin D, a pre-hormone, go far beyond regulating bone metabolism, playing a crucial role in the immune system and in maternal and fetal genetic modeling, with the ability to regulate genome expression, activating or deactivating specific genes [15,17].

The Human Genome Project has led to the emergence of knowledge in genetics and genomics that has caused profound changes in the current paradigm, and the integration and management of this knowledge in care is essential [18,19,20,21]. Currently, the study of genetic polymorphisms in the identification of genetic and epigenetic risk factors at an individual level is a scientific revolution in nutrition science, with the aim of developing personalized nutrition approaches integrated into an ecosystem of empowerment and health promotion [15,16].

In particular, the Portuguese population has a higher prevalence of genome alterations that confer a lower capacity to produce vitamin D from sunlight exposure. These genetic characteristics are present in about 19% of the population, representing prevalence four times higher than the European average (19% vs. 4.75%). The prevalence of these specific polymorphisms leads to a higher predisposition to vitamin D deficiency [22].

Maternal vitamin D levels have never been the subject of a longitudinal study in Portugal involving two distinct collection points. The relevance of vitamin D levels and its potential impact on maternal and infant health outcomes, particularly in reducing avoidable premature births, were fundamental to the design of this multicenter observational study. The planned research project involves implementing multicenter recruitment centers, with a minimum sample size of 1800 participants with the goal of investigating the effects of maternal vitamin D levels on prematurity. This sample size was calculated based on the prevalence, in Portugal, of prematurity as the primary outcome variable. The recruitment and data collection are already underway in healthcare organizations across the Alentejo region, one of the sunniest areas in the country. It includes both primary and specialized healthcare units, with local doctors and nurses actively collaborating in the process.

It is important to highlight that our pilot study was specifically designed to assess the feasibility of the recruitment process and the planned procedures for the upcoming large-scale multicenter observational study. While it was not intended to evaluate the effects of maternal vitamin D levels on prematurity, this crucial question will be thoroughly addressed in the larger study, building on the foundations established by this pilot phase.

In this pilot study, the feasibility assessment included validation of data collection instruments, evaluation of recruitment potential, and the adequacy of the coding process and data organization, processing, and analysis. Furthermore, the feasibility of inter-organizational collaboration was also assessed. The evaluation of these criteria will lead to a secondary assessment that will inform the decision to proceed with the planned study [23,24].

## 2. Methods

### 2.1. Design

This observational pilot study follows the protocol of a more ambitious research project, which includes a large-scale multicenter prospective cohort study. The main objective of the project is to measure vitamin D levels during pregnancy and assess their impact on outcomes related to prematurity. We aim to determine how the prevalence of hypovitaminosis D affects prematurity outcomes. This research requires monitoring maternal 25(OH)D levels through prenatal and postnatal surveillance, incorporating this biomarker into routine blood collections. Biometric and biochemical data will be collected at two distinct time points: the first during the initial prenatal surveillance appointment, and the second collection up to 48 h after birth during the hospitalization period. This pilot study will focus on assessing the feasibility of the recruitment and data collection procedures.

The large-scale multicenter observational study will also assess maternal vitamin D deficiency and its associated factors. It aims to identify genetic polymorphisms related to the vitamin D pathway and assess their associations with adverse clinical outcomes related to prematurity. This involves collecting saliva samples and conducting genetic analyses to study polymorphisms in seven genes, integrating the analysis of 18 genetic variants involved in vitamin D metabolism. These procedures will proceed once funding has been secured. However, the planned process involves a request for genetic analysis made by the local medical collaborator and saliva collection by the participant—a non-invasive method—to investigate genetic polymorphisms related to vitamin D. Saliva samples from pregnant women will be collected during hospitalization, either before or after delivery, under the supervision of a healthcare professional. For all samples, the transport, analysis, and disposal of genetic material will be carried out in partnership with a certified genetic testing laboratory. After collection, using an Isohelix Buccal swab kit including stabilization buffer, the biological sample will be stored at room temperature and sent to the laboratory by the local collaborator via standard mail in a “triple-packed” model. The Isohelix Buccal swab kit is designed to stabilize DNA in saliva samples for extended periods. According to the manufacturer and the genetic testing laboratory, DNA can be preserved at room temperature (15–25 °C) for up to 12 months. After DNA extraction and if long-term storage is required, the samples will be frozen at −20 °C. The sample will be transported, three days maximum, in a sealed envelope, protected within a second padded envelope. After analysis, the anonymous report, identified by a code, will be delivered to the healthcare professional responsible for the data.

This research project entitled VitDTracking was registered on 4 March 2024 in the ClinicalTrials.gov database (Identifier: NCT06292195) https://clinicaltrials.gov/study/NCT06292195. The pilot follows the Strengthening the Reporting of Observational Studies in Epidemiology (STROBE) guidelines [25].

### 2.2. Recruitment Process

The pilot study involved the implementation of multicenter recruitment centers across three public health units in the Alentejo region (north, south, and center), identified in this paper as ULSNA, ULSBA, and ULSAC. Local collaborators, health professionals, doctors and nurses conducted recruitment. In this pilot, 30 participants were enrolled. The pilot study was conducted in accordance with eligibility criteria established for the large-scale investigation. As inclusion criteria, it was defined that participants should be pregnant women living in the Alentejo region, aged 16 years or older. Pregnant women with language barriers or communication difficulties were excluded, including those who had difficulty speaking, reading, and writing Portuguese or understanding basic information necessary for decision-making.

Recruitment began with the distribution of the study’s information leaflet to eligible participants. Pregnant women who agreed to participate were given a consent form in duplicate (one for the Project File and another for the participant) and a coded questionnaire. The registration and coding of participants in the cohort were carried out by the local collaborator, following the return of the signed consent form and completed questionnaire. The next step involved the local collaborating doctor requesting the measurement of 25(OH)D levels for the recruited participants. The request was made using the SClínico software program (version number SC 2.8.2), which supports the Portuguese National Health Service. To facilitate the logistical aspects of recruitment, a Research Support File (RSF) was created for each unit. The RSF contains the following items: institutional authorizations, Ethic Committee approval, the research protocol, participant registration form, data collection instruments (questionnaire and clinical records), informed consent, information flyer, and the coding process.

### 2.3. Coding Process

A unique code was assigned to each unit and conveyed to the local collaborators. Two files were created for the purpose of participant registration. One file containing the identification of participants (name and/or patient number) and a code assigned in ascending order of participation. The second file includes the code of each participant along with the remaining collected data. To facilitate this process, a Data Collection Notebook (DCN) was created, encompassing all the parameters to be recorded by the local collaborator. Each DCN was coded with the same code assigned to the participant in the registration document and the questionnaire.

### 2.4. Data Collection Instruments

The principal data collection instruments were the questionnaire and the clinical records. The questionnaire, comprising 37 main questions, aimed to characterize the cohort through sociodemographic data, lifestyle factors, obstetric, and health history. Given the size of the cohort and to ensure the anonymity of the participants, the categorical variables can be viewed in the Supplementary Information, but without the absolute and relative frequency values. Data obtained included age, ethnicity, employment status, pre-pregnancy weight and height, sleep habits, smoking and alcohol habits, physical exercise practices, duration of physical activity, sun exposure, diet type, frequency of consumption of vitamin D-rich foods, skin type, parity, previous abortions, history of preterm births, and vitamin D supplementation.

The pre-pregnancy body mass index (BMI) was calculated based on the values indicated in the questionnaire by the participant. This was performed by dividing pre-pregnancy weight by height squared [BMI = Weight (kg)/Height^2^ (m)]. The pre-pregnancy BMI was classified according to World Health Organization guidelines [26]: underweight (<18.5 kg/m^2^), normal weight (18.5–24.9 kg/m^2^), overweight (25–29.9 kg/m^2^), and obesity (≥30 kg/m^2^). Skin type was categorized following the Fitzpatrick phototype scale [27]: Type I—Pale white skin, red or blonde hair, blue or green eyes, freckles; Type II—Fair skin, red or blonde hair, blue, light brown, or green eyes; Type III—Darker white skin, any hair or eye color; Type IV—Light brown skin; Type V—Brown skin; Type VI—Dark brown or black skin. The sun exposure area was categorized as follows: face and hands (10%), face, arms, and hands (20%), and face, arms, hands, and legs (35%) when wearing shorts and a short-sleeved t-shirt [28,29].

The DCN integrates the data collected from the questionnaire with 18 maternal and 13 neonatal unique parameters obtained from clinical records, focusing on maternal–infant health outcomes. From these parameters, 10 were selected to be measured multiple times to assess the quality of data collection. These 31 clinical parameters include two 25(OH)D assessments: one during the prenatal period and a second measurement postpartum. In this pilot study, only one assessment of this biomarker was considered for correct completion, thus adjusting the total number of clinical parameters to 30. The collection of clinical characteristics focused on indicators of the current pregnancy’s progression and the maternal–infant outcomes. Data collected included medication taken during pregnancy, maternal vitamin D levels, prenatal complications, gestational weight gain (GWG), type of delivery, and adverse clinical effects in the postpartum period. GWG was calculated by the difference between the weight measured before delivery at hospital admission and the pre-pregnancy weight reported by the participant in the questionnaire. GWG ranges were classified according to the Institute of Medicine recommendations [30]: insufficient (<11 kg), adequate (11–16 kg), and excessive (>16 kg). A systolic blood pressure (SBP) ≥ 140 mmHg or a diastolic blood pressure (DBP) ≥ 90 mmHg, in two separate measurements taken at least 6 h apart, defined gestational hypertension [31]. Blood pressure was measured at enrollment. The diagnosis of gestational diabetes was made through the oral glucose tolerance test (OGTT) between 24 and 28 weeks of gestation. Clinical pathology technicians performed the OGTT in the morning, after an 8–12 h fast, with the pregnant woman at rest, repeated after 1 h and 2 h. Fasting OGTT values ≥92–125 mg/dL, ≥180 mg/dL after 1 h, or 153–199 mg/dL after 2 h confirmed the diagnosis [32].

In particular, data pertaining to neonatal characteristics were collected, including gestational age at birth (GAB), sex, weight, length, and head circumference at birth, Apgar score at 1 and 5 min, admission to the neonatal intensive care unit (NICU), days of hospitalization, and neonatal complications. GAB was calculated based on the gestational age estimated by obstetric ultrasonography and/or the last menstrual period date. Preterm birth has been defined as birth occurring before 37 completed weeks of gestation and term birth as birth ≥ 37 weeks of gestation [33]. Newborn anthropometric measures at birth were classified according to the WHO Child Growth Standards [34]. Birth weight for GA: extremely low birth weight (<1000 g), very low birth weight (1000–1499 g); low birth weight (1500–2499 g); insufficient weight (2500–2999 g); normal weight (3000–3999 gr) and macrosomia (≥4000 g). Length for GA (short length < 50 cm and normal length ≥ 50 cm) and head circumference for GA (small head circumference < 35 cm and normal head circumference ≥ 35 cm).

The questionnaire and clinical parameters used in this pilot study were developed based on a review of the literature. Biometric and biochemical data collection was performed through blood samples integrated into routine perinatal surveillance. The usual parameters were supplemented with the serum marker 25(OH)D, considered the most reliable indicator of body vitamin D stores.

### 2.5. Method for Assessing 25(OH)D Levels in Maternal Serum

In the pilot study, since recruitment began simultaneously in primary and secondary healthcare units, maternal blood samples were collected and analyzed for 25(OH)D levels at a single time point for each participant. The 15 pregnant women recruited during the prenatal period had their samples collected immediately after enrolling in the cohort. For the 15 pregnant women recruited during admission to the obstetrics service, the samples were collected within the first 48 h postpartum in all three healthcare units. It was very important to ensure that it was possible to carry out this vitamin D measurement within 48 h, since there are several scientific publications that show a strong association between maternal and newborn vitamin D levels in this time interval [35,36,37].

The collections were performed by clinical pathology technicians working at the recruitment centers. The quantitative determination of total serum 25(OH)D concentrations was performed using the ELISA (Enzyme-Linked Immunosorbent Assay) method, which is standardized across all three healthcare units to ensure the consistency of laboratory results. Depending on the serum 25(OH)D levels, pregnant women were classified according to the Endocrine Society reference cut-off levels: deficiency < 20 ng/mL, insufficiency 20–29 ng/mL, and sufficiency ≥ 30 ng/mL, considered normal [38].

### 2.6. Outcomes

Considering the main objective of the pilot study, the key outcomes are related to evaluating the feasibility of the processes and the capacity of the participating recruitment centers. For this evaluation, indicators and success criteria were defined, as presented in Table 1.

The success criteria were defined based on the target sample size of approximately 1000 eligible participants, the size of the potential global cohort, and the established recruitment timeline. The multicenter observational study will tap into global recruitment efforts, aiming to enroll around 1800 pregnant women to ensure the minimum eligible sample size is met. Success metrics were set at challenging yet achievable levels, ensuring each center sustains robust recruitment and high-quality data collection while aligning with the study’s scale and objectives.

To determine the sample size required for the large-scale study, the following factors associated with the percentages obtained in this implementation pilot have been considered: the global recruitment rate (73.17%), the eligible recruitment rate (88.2%), the refusal rate (11%), and the prevalence of prematurity in Portugal (10%). The primary objective was to ensure a final sample including a statistically significant number of participants exhibiting preterm births. Starting with a population of 1800 pregnant women and considering a global recruitment rate of 73.17%, an eligible recruitment rate of 88.2%, and an acceptance rate of 89%, we expect to collect data from at least 1000 participants (1800*0.7317*0.882*0.89). Since the prematurity rate in Portugal is around 10%, we expect to observe at least 100 participants who meet the preterm criterion.

### 2.7. Statistical Analysis Plan

Statistical analysis will be conducted using IBM SPSS^®^ statistical software (version 27). More sophisticated data analysis is also planned by using the FLOWER framework to implement federated learning to integrate the data from the different centers, ensuring the security and privacy of the data [39]. Initially, a descriptive analysis of categorical and numerical variables will be performed. Measures of central tendency: mean, median, quartiles, maximum, and minimum, and measures of dispersion: range, interquartile range, standard deviation (SD), and variance, as well as skewness to describe the asymmetry of the distribution, will be used to describe numerical variables. Frequency and percentage will be used to describe categorical data. The normality of numerical data will be assessed using the Kolmogorov–Smirnov test. If the normality assumption is met, Student’s *t*-test, including the independent *t*-test, will be used to compare 25(OH)D levels between participants with different characteristics, pregnancy indicators, and neonatal outcomes. If the normality assumption is not met, the non-parametric Mann–Whitney test will be used [40].

In the bivariable analysis to evaluate differences between groups (with and without hypovitaminosis D) concerning categorical variables, Pearson’s chi-square test of association will be used. If up to 25% of the expected frequencies are less than 5, Fisher’s exact test will be used. To evaluate the strength of the association, the Odds Ratio (OR), a measure of relative effect, will be calculated with a 95% confidence interval (CI). A statistical significance level of 5% (*p* < 0.05) will be considered. Bonferroni multiple test correction will also be used. A multivariable logistic regression analysis will be performed to explain the association of maternal vitamin D deficiency risk factors with adverse maternal–infant clinical outcomes. Variables with a *p*-value ≤ 0.20 in the bivariate analysis will be selected for the regression model using the calculation of the *p*-value, chi-square, and OR. Several regressions with adjusted OR values for each model will be performed until reaching the final model. To test the regression model, the Hosmer–Lemeshow test and the Omnibus test will be used [40]. Lastly, other machine learning techniques, like decision trees, will be used in order to define a risk classifier.

### 2.8. Ethical Aspects

The protocol was developed in accordance with the ethical principles of the Declaration of Helsinki and in compliance with the Good Clinical Practice guidelines [41]. The project was approved by the Ethics Committee of the University of Évora (registration number 22094). Subsequently, the protocol was submitted to the Ethic Committees (HEC) from the recruitment healthcare institutions. The project is identified with the following references: No. 067/22; No. 13/2023; No. 86/2022; and No. 2, EDOC/2022/50917. Recruitment was voluntary following the participants’ expression of interest and the signing of informed consent. Confidentiality was assured, and participants were informed that they could withdraw from the study at any time if they wished.

## 3. Results

### 3.1. Recruitment

**Figure 1:**
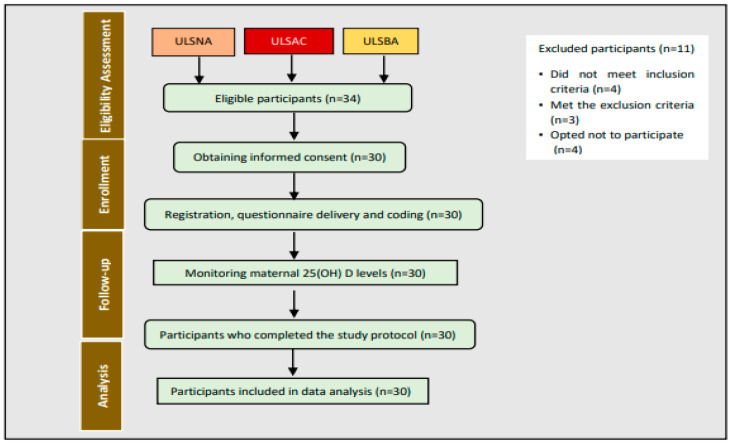
Pilot study protocol flowchart.

The participating centers demonstrated adherence to the specified recruitment timeline. A total of 41 participants have been approached to participate in the project. Of these participants, seven were excluded due to inclusion or exclusion criteria, leaving 34 participants as possibly eligible. Of these eligible participants, four opted not to sign the informed consent form. A total of 30 eligible participants were enrolled between June and October 2023 and agreed to participate in the study either during prenatal monitoring or upon admission to the Obstetrics service. The follow-up period extended from the time of cohort enrollment until the completion of all study procedures (Figure 1). This recruitment process shows that it was possible to guarantee a global recruitment rate of over 70% and an eligible recruitment rate of over 75%.

### 3.2. Assessment of Feasibility Criteria

Regarding the feasibility assessment, it can be observed in Table 2 that all defined criteria were met. The acceptability of study processes by the centers was evaluated through reports prepared by local collaborators. These reports identified issues or challenges such as protocol deviations and their respective resolutions. The reports are presented as follows:

- Doctors integrated into the multidisciplinary team do not request 25(OH)D testing from recruited participants, primarily citing two reasons: the high cost of the vitamin D reagent and a lack of understanding regarding the project’s relevance or the importance of testing in prenatal and postnatal monitoring.

Resolution: The costs of serum 25(OH)D measurement were covered by the Portuguese National Health Service and authorized by the administrative boards of all the Local Health Units that participated in the recruitment process. When the doctor was unavailable, the nursing team or the coordinator requested the testing from another medical collaborator, either on the same day or the following day. Training sessions on the importance of vitamin D in this context and the need to measure this parameter for the project were reinforced.

- Difficulty integrating the request for 25(OH)D testing into routine prenatal surveillance parameters.

Resolution: Hospital management and IT services were engaged to include this parameter in routine prenatal and postnatal surveillance at the recruitment centers.

- Difficulty in requesting two measures of 25(OH)D.

Resolution: The main issue is related to the follow up measure because most of the participants gave birth in a different healthcare facility than the one where they were followed during pregnancy. This issue is not related to the difficulty of performing the test. It was resolved by mapping the participant’s identification number from the initial contact with the project (when enrolled) to the new identifier assigned during the follow-up process. Other mitigation strategies were added. We reinforced closer and more frequent monitoring of the recruitment centers through additional clarification and motivation sessions directed at the healthcare teams. We increased the number of healthcare professionals directly involved in the project and assigned these professionals liaison roles. During the prenatal recruitment phase, it was requested that the phrase “integrated into the VitDTracking project” or simply “VitD Project” be added to the Pregnancy Booklet. This mention of the project helps ensure the follow-up of the participant in the postpartum period, with the request for the second collection point. These strategies are providing positive results in the large-scale study already in the implementation phase.

- Failure to record some clinical parameters included in the project’s data collection protocol.

Resolution: Meetings were held at recruitment centers with doctors and nurses, with the aim of reinforcing the importance of having all the defined parameters assessed and recorded, increasing commitment to the project. An effort was made, in partnership with healthcare professionals, to understand why this failure is predominant in certain clinical parameters such as C-reactive protein (CRP) level, oral glucose tolerance test (OGTT), systolic and diastolic blood pressure (SBP and DBP), maternal weight before delivery, birth length, and head circumference at birth.

- Platform failures preventing visibility of the testing request in the system.

Resolution: Enhanced emphasis on the request in the observation field.

- Issues with requesting testing on weekends in the SClínico system (only urgent profiles available on weekends).

Resolution: Collaboration was sought from clinical pathology technicians to conduct non-urgent collections over weekends.

- Participants took both signed informed consents home.

Resolution: Healthcare professionals contacted the participant who returned to the unit to deliver one of the signed informed consents.

These resolutions effectively addressed the challenges identified during the study, ensuring adherence to protocol and smooth operation of procedures at the recruitment centers.

### 3.3. Characterization of the Cohort

In the pilot study, the comparative analysis between the groups with and without hypovitaminosis D was not carried out due to the low size of the cohort. As the primary objective of the pilot study is to assess the feasibility of the protocol rather than to determine statistical significance, only the characterization of the cohort using univariable analysis is presented. The numerical variables were described using the mean, median, quartiles, maximum, minimum, range, interquartile range, standard deviation, variance, and asymmetry (Supplementary Table S1). Categorical variables were described using frequency and percentage. However, as previously mentioned, to ensure the anonymity of the participants, the categorical variables can be viewed, but without the absolute and relative frequency values (Supplementary Tables S2 and S3).

In the sample of 30 pregnant women, 29 neonates were born, with one miscarriage occurring before 24 weeks. The average age of the participants is over 30 years. The mean 25(OH)D level was below 30 ng/mL, with a recorded minimum value of 7 ng/mL. The reported average value was obtained by considering that the pregnant women were globally in the perinatal period. In this pilot, it was possible to see how difficult it was to obtain the two vitamin D measurements required for the project’s objectives. Only one of the participants had their vitamin D measured at two different points: one prenatal and one postnatal. This procedural difficulty has already been corrected as described in the subsection “Assessment of feasibility criteria”. Notably, the mean pre-gestational BMI was 25.1kg/m^2^, falling into the overweight category. Regarding the neonates, the mean length at birth (48 cm) and head circumference (34.6 cm) were below the normal values of 50 cm and 35 cm, respectively. All neonates were born with an Apgar score of 8 or higher.

The analysis of categorical data from this cohort of 30 participants revealed a prevalence of hypovitaminosis D of 63.3% (19 pregnant women), during the perinatal period. In the group of 16 pregnant women whose samples were collected during pregnancy, 43.8% (7) exhibited hypovitaminosis D. Of these 7 pregnant women, 42.9% (3) were taking vitamin D supplementation.

In the group of 15 participants whose blood samples were collected postpartum, the percentage of hypovitaminosis D was 86.7% (13). Notably, of these 13 women, 76.9% (10) took vitamin D supplements during pregnancy.

Regarding sociodemographic characteristics, the majority of the pregnant women (51.7%) were aged 25 to 34 years, 43.3% had higher education, 70% were married or in a stable union, and 83.3% were Portuguese, of white race, and employed. According to the Fitzpatrick phototyping scale for skin type, 53.3% of the pregnant women reported having type III skin. Concerning habits and lifestyle, 93.3% of the participants followed a Mediterranean diet, 40% had smoking habits, 36.7% were passive smokers, 43.3% had alcohol habits, 40% engaged in physical exercise, and 60% of the pregnant women habitually slept seven or fewer hours. Pre-gestational BMI showed that 46.7% had values above normal, with 30% being overweight and 16.7% being obese. The most consumed vitamin D-rich foods on a weekly basis were milk and dairy products. During outdoor activities, 60% of the participants usually exposed 20% of their body area. Regarding sunscreen use, 60% reported applying it only in the summer, which includes the recruitment period.

In terms of obstetric determinants, 93.3% of the participants reported conceiving naturally, 30% were in their first pregnancy, 40% were nulliparous, 40% reported previous miscarriages, and 6.7% had preterm births. Concerning medication use, 70% of the participants stated they were taking medication during pregnancy. The medications mentioned included those related to pregnancy such as Nausefe, acetylsalicylic acid, Lovenox, and supplements of iodine, iron, folic acid, potassium iodide, vitamin D, as well as medications for specific conditions like Copaxone, sertraline, and quetiapine. Of the 29 births, 62.1% (18) were by dystocia, of which 72.2% (13) were by cesarean section. Among the 30 participants, 43.3% (13) experienced adverse clinical effects, with gestational diabetes having the highest prevalence at 31.3%, followed by IUGR at 18.8%. It should be noted that of the 21 pregnant women with weight assessed before delivery, 26.7% registered excessive gestational weight gain.

Regarding neonatal variables, of the 29 live-born neonates, 58.6% were male, and 96.6% were born at term. There was one preterm birth at 34 weeks who was hospitalized in the NICU for 5 days. Low birth weight (LBW) was present in 10.3% of the neonates, 79.3% had a short length at birth, and 51.7% had a small head circumference. Two neonates (6.9%) had adverse clinical effects, including respiratory distress syndrome requiring oxygen therapy and feeding intolerance during hospitalization.

## 4. Discussion

The results revealed that the pilot study successfully met all predefined feasibility criteria. The pilot demonstrated strong recruitment and participation outcomes, with a global recruitment rate of 73.17% and an eligible recruitment rate of 88.2%. These rates indicate a high level of interest and willingness among eligible participants to join the study. The low refusal rate of 11% further underscores the feasibility of recruiting participants for similar future studies. Adherence to the study protocol was excellent, as evidenced by the 100% adherence and retention rates. This suggests that the study design and procedures were well-accepted by participants and that the study environment was conducive to maintaining strong participant engagement and compliance over time. The completion rate, also 100% when considering the initial protocol’s single evaluation of maternal 25(OH)D, further confirms the feasibility of the study design. While the questionnaire comprehension rate of 86.6% indicates that most participants understood the survey content, it also highlights a potential area for improvement, suggesting that the large study could benefit from revising the questionnaire to increase clarity.

The participating centers proved to be well-prepared, with all centers meeting the required installed capacity and showing over 90% compliance with the study protocol. This reflects the ability of the centers to conduct the study effectively and may serve as a benchmark for future multicenter studies. The clinical record completion rate of 93.11% is encouraging, although it showed partial completion of 148 items across seven parameters (CRP, OGTT, SBP, DBP, maternal weight before delivery, birth length, and head circumference at birth). The recurring failure to record certain specific protocol parameters was one of the issues identified during the pilot study. To address this, additional meetings were held at the recruitment centers with physicians and nurses to emphasize the importance of evaluating and recording all defined parameters.

All local collaborators reported having sufficient time to perform the required procedures, and all solvable challenges were addressed promptly and effectively. Despite the partial completion of specific parameters, the overall feasibility of the study was confirmed, with high adherence to the protocol and successful data collection across multiple centers.

The data obtained from the Alentejo region also revealed a concerning prevalence of hypovitaminosis D among participants. Specifically, in the group of 15 participants whose blood samples were collected within the first 48 h postpartum, the percentage of hypovitaminosis D was very high (86.7%). It is also noteworthy that within this group, where 13 participants had hypovitaminosis D, 76.9% (10) took vitamin D supplements during pregnancy. These findings are consistent with the literature indicating that maternal vitamin D deficiency is common even in areas with high sun exposure and remains high when supplementation is not adjusted to individual needs [10,11,14,38].

In our pilot study with 30 participants, it was not possible to perform a bivariate analysis to assess a dose–response relationship due to the limited sample size. However, a larger-scale study involving 1000 pregnant women would provide sufficient statistical power to investigate the relationship between the dose of vitamin D supplementation during pregnancy and serum levels of vitamin D. For the same reason of the limited sample, the associations between maternal vitamin D levels and prematurity were not analyzed. However, one premature birth at 34 weeks was recorded. Maternal blood collection for the measurement of 25(OH)D was performed in the prenatal period at 28 weeks of pregnancy, and the value was 36 ng/mL. The second collection to assess this parameter after birth was missing, confirming the importance of assessing vitamin D at two different points defined in the protocol, allowing for the tracking of this measure in the postnatal period. In the questionnaire, this pregnant woman reported a previous history of miscarriage and a preterm delivery at 33 weeks. This cohort also recorded a miscarriage at 17 weeks, with a 25(OH)D value of 21 ng/mL measured one week before the occurrence of this outcome. It should also be noted that this participant reported a previous history of prematurity (preterm birth at 36 weeks).

The lack of longitudinal data on maternal 25(OH)D levels was a limitation of the pilot study. Blood sampling for monitoring this marker at two distinct points, during prenatal surveillance and postpartum, will be integrated into the protocol of the larger study, undoubtedly contributing to a more comprehensive understanding of variations in maternal vitamin D levels and associations with maternal–infant outcomes. This knowledge will be crucial for designing and developing personalized public health strategies for the Portuguese population, including appropriate vitamin D supplementation, especially during and after pregnancy.

## 5. Conclusions

The pilot study, which included 30 pregnant women, validated the feasibility of the recruitment methodologies and procedures, demonstrating that conducting a large-scale multicenter observational study on the effects of maternal vitamin D levels, involving a sample of 1000 participants is achievable. Feasibility also encompasses the acceptability by recruitment centers, collaborators, and participants. Evaluating these criteria was crucial for a secondary assessment, which involved the decision to proceed with the planned study.

The pilot results also underscore the importance of collaboration among different healthcare units and professionals, ensuring that research protocols are strictly followed and that participants receive continuous and effective monitoring of vitamin D levels during and after pregnancy. Identifying logistical and methodological challenges by collaborators that could hinder the conduct of the research will contribute to increasing the likelihood of success in implementing the main study.

In summary, this pilot study not only validated the need and relevance for further research on maternal vitamin D levels in Portugal but also provided an effective model for implementing such studies. The findings can inform public health policies and clinical practices in creating more effective and personalized supplementation programs, improving maternal and neonatal outcomes, and promoting overall population health.

## Figures and Tables

**Table 1:** Assessment of process feasibility and recruitment center viability in resources and data management.

Objectives	Indicators	Success Criteria
**To Assess Process Feasibility**	Global recruitment rate (number of participants recruited/total number of potential participants × 100)	Global recruitment rate of at least 70% of potential participants
	Recruitment rate after eligibility (number of participants recruited/number of eligible potential participants × 100)	Eligible recruitment rate of at least 75% of eligible potential participants
	Refusal rate (number of participants who refused to participate in the study/total number of eligible potential participants × 100)	Refusal rate less than 25% among eligible potential participants
	Adherence rate (number of participants who start study procedures/number of participants recruited × 100)	Adherence rate of at least 80% of recruited participants who start study procedures
	Retention rate (number of participants who complete the procedures/number of participants recruited × 100)	Retention rate of at least 90% of recruited participants who complete study procedures
	Completion rate (number of participants who completed all procedures/number of participants who started study procedures × 100)	Completion rate of at least 80% of participants who started the study and completed all procedures
	Questionnaire comprehension rate (number of questionnaires correctly completed/total number of questionnaires distributed × 100)	Ensure that at least 85% of the participants provide complete responses to the study questions (without missing or incomplete answers).
**To assess the feasibility of recruitment centers in resources and data management**	Installed capacity of recruitment centers	All centers have necessary resources (human, financial, materials, and equipment) for effective study conduct
	Acceptability of study processes	At least 90% compliance with study protocol (procedures and responsibilities, not considering the questionnaire) by recruitment centers
	Availability of time for local collaborators	At least 70% of healthcare professionals report sufficient time for necessary procedures under the Research Protocol
	Resolution of human resource and data management issues	At least 75% timely and effective resolution of challenges in human resource and data management (collection, coding, storage) identified by centers

**Table 2:** Assessment of feasibility criteria.

Success Criteria	Assessment of Feasibility Criteria
Global recruitment rate of at least 70% of potential participants	Global recruitment rate = 73.17% (30/41 × 100)
Eligible recruitment rate of at least 75% of eligible potential participants	Eligible recruitment rate = 88.2% (30/34 × 100)
Refusal rate less than 25% among eligible potential participants	Refusal rate = 11% (4/30 × 100)
Adherence rate of at least 80% of recruited participants who start study procedures	Adherence rate = 100% (30/30 × 100)
Retention rate of at least 90% of recruited participants who complete study procedures	Retention rate = 100% (30/30 × 100)
Completion rate of at least 80% of participants who started the study and completed all procedures	Completion rate = 100% 30/30 × 100 (considering only one vitamin D evaluation which was the initial protocol)
Ensure that at least 85% of the participants provide complete responses to the study questions (without missing or incomplete answers)	Questionnaire comprehension rate = 86.6% (26/30 × 100)
All centers have necessary resources (human, financial, materials, and equipment) for effective study conduct	Participating centers have the required installed capacity to implement the study
At least 90% compliance with study protocol (procedures and responsibilities not considering the questionnaire) by recruitment centers	The centers complied with the study protocol by more than 90% Total 30 clinical parameters—considered only an assessment of maternal 25(OH)D levels Clinical records completion rate: (838/900) × 100 = 93.11% 30 participants × 30 parameters = 900 expected items 23 parameters × 30 participants = 690 items completed Total items completed: 690 (fully completed) + 148 (partially completed) = 838 Items partially completed = 148 CRP: 7; OGTT: 24; SBP: 21; DBP: 21; Maternal weight before delivery: 21; Birth length: 27; Head circumference at birth: 27
At least 70% of healthcare professionals report sufficient time for necessary procedures under the Research Protocol	All local collaborators reported having enough time to carry out the procedures
At least 75% timely and effective resolution of challenges in human resource and data management (collection, coding, storage) identified by centers	All the problems/challenges that could be solved were resolved in a timely and effective manner. However, some parameters were not recorded in the clinical file, such as maternal weight before delivery, blood pressure, oral glucose tolerance test, among others

## Data Availability

The datasets generated and analyzed during this study were stored in accordance with the University of Évora’s data storage policy. Access to the data will be possible at the end of the VitDTracking project, under the approval of the Data Access Committee.

## Supplementary Materials

The following supporting information can be downloaded at: https://www.mdpi.com/article/10.3390/nu17071160/s1, Supplementary Table S1: Statistical values of numerical variables; Supplementary Table S2: Baseline characteristics of the cohort based on the questionnaire parameters; Supplementary Table S3: Baseline characteristics of the cohort based on clinical record parameters.
