# Supplementary material for: Effects of Maternal Vitamin D Levels on Prematurity: Feasibility Study in a Multicenter Observational Pilot
Source: Nutrients. 2025 Mar 27;17(7):1160. doi: 10.3390/nu17071160 (PMC11990355; doi:10.3390/nu17071160)
Supplement: Supplementary file 1 [file nutrients-17-01160-s001.zip › nutrients-3524846-supplementary.pdf]

EFFECTS OF MATERNAL VITAMIN D LEVELS ON PREMATURITY: FEASIBILITY STUDY IN A MULTICENTER OBSERVATIONAL PILOT

Olívia Barbosa[1,2,3], Ana Teresa Freitas[4,5], Marta P. Silvestre[6,7], André Moreira-Rosário[6,7], Pedro Aguiar[8], Ana Isabel Régua[9], Tatiana Madaleno[10], Manuela Almeida[11], Dulce Cruz[1,2]

[1] Comprehensive Health Research Centre (CHRC), Lisboa, Portugal
[2] Universidade de Évora, Évora, Portugal
[3] Neonatal Intensive Care Unit, Unidade Local de Saúde do Alentejo Central (ULSAC), Évora, Portugal
[4] Instituto de Engenharia de Sistemas e Computadores - Investigação e Desenvolvimento em Lisboa (INESC-ID), Lisboa, Portugal
[5] Instituto Superior Técnico, Universidade de Lisboa, Lisboa, Portugal
[6] NOVA Medical School, Universidade NOVA de Lisboa, Lisboa, Portugal
[7] CINTESIS@RISE, NOVA Medical School, Universidade NOVA de Lisboa, Lisboa, Portugal
[8] NOVA National School of Public Health**,** Universidade NOVA de Lisboa, Lisboa, Portugal
[9] Unidade Local de Saúde do Baixo Alentejo (ULSBA), Beja, Portugal
[10] Unidade Local de Saúde do Norte Alentejo (ULSNA), Portalegre, Portugal
[11] Unidade Local de Saúde do Alentejo Central (ULSAC), Évora, Portugal

Corresponding author:

Olívia Barbosa, MSc, PhD Student

Comprehensive Health Research Centre, Lisboa; Universidade de Évora; Neonatal Intensive Care Unit, Unidade Local de Saúde do Alentejo Central (ULSAC), Évora, Portugal

Tel.: 00351 966056178

Email: d52901@alunos.uevora.pt

Supplementary Table S1. Statistical values of numerical variables

Supplementary Table S2. Baseline characteristics of the cohort based on the questionnaire parameters

Supplementary Table S3. Baseline characteristics of the cohort based on clinical record parameters

Table S1. Statistical values of numerical variables

	N	Mean	Median	Variance	SD	Minimum	Maximum	Range	1st Quartile	2nd Quartile	3rd Quartile	Interquartile Range	Asymmetry
**Maternal age (years)**	30	30.5	30	44.5	6.7	17	42	25	27.8	30	35.2	8	-0.2
**Perinatal 25(OH)D (ng/mL)**	30	25.8	20.3	280.9	16.7	7	84	77	13	20.3	32.9	20	1.7
**Prenatal 25(OH)D (ng/mL)**	16	27.9	31	161.1	12.7	7	54	47	17.2	31	36.8	20	0.03
**Postnatal 25(OH)D (ng/mL)**	15	23	16	402.7	20	10	84	74	12	16	20	8	2.5
**Pre-gestational BMI (kg/m^2)**	30	25.1	23.8	30.6	5.5	17	40	24	20.8	23.8	29.4	9	0.9
**Gestational age at blood sampling (weeks)**	30	33.5	38	62.8	7.9	16	41	25	30.2	38	39.2	9	-1.09
**Systolic blood pressure (mmHg)**	21	120.5	118	252.8	15.9	93	168	75	110	118	130	20	1.2
**Distolic blood pressure (mmHg)**	21	69.9	72	132.8	11.5	50	102	52	60.5	72	75	15	0.7
**OGTT Fasting (mg/dL)**	24	75.8	77	115.8	10.8	54	92	38	65	77	84.5	19	-0.4
**OGTT after 1h**	20	129.8	132.6	1047.1	32.4	71.7	182	110.4	98.7	132.6	155.6	56.3	-0.06
**OGTT after 2h**	20	103.6	106	787.7	28.1	54	155.1	101.1	77.5	106	122.4	44.9	-0.09
**C-Reactive Protein (mg/L)**	7	5.5	3.6	18.9	4.3	2	14.3	12.3	2.1	3.6	6.9	4.8	1.6
**Gestational weight gain (Kg)**	21	13.2	12.7	45	6.7	0	24	24	7.5	12.7	19	12	-0.13
**Gestational age at birth (weeks)**	29	39	39	1.7	1.3	34	41	7	39	39	40	1	-2.15
**Birth weight (gr)**	29	3120.1	3220	141784.5	376.5	2210	3670	1460	2860	3220	3395	535	-0.7
**Length at birth (cm)**	27	48	48	3.2	1.8	44	51	7	46.5	48	49	2.5	-0.7
**Head circumference (cm)**	27	34.6	34.5	2.5	1.6	31.5	38	6.5	33.3	34.5	36	2.7	0.02
**Apgar 1st minute**	29	9.3	9	0.4	0.6	8	10	2	9	9	10	1	-0.24
**Apgar 5th minute**	29	9.9	10	0.07	0.3	9	10	1	10	10	10	0	-3.6
**Neonatal hospitalization (days)**	29	2.7	3	0.5	0.7	2	5	3	2	3	3	1	1.25

Table S2. Baseline characteristics of the cohort based on the questionnaire parameters

		Number	Percentage (%)
Maternal characteristics (n=30)			
**Age Group** (years)	16-17		
	18-20		
	21-24		
	25-34		
	35-44		
**Nationality**	Portuguese		
	Other		
**Ethnic Origin**	White		
	Black		
	Asian		
	Gypsy		
	Other		
**Latitude**	Portalegre		
	Évora		
	Beja		
**Residence**	Urban		
	Rural		
**Type of House**	Apartment		
	Villa		
	Other		
**Marital Status**	Single		
	Married		
	Stable union		
**Education**	Basic		
	Secondary		
	Professional course		
	Higher education		
**Employment**	Employee		
	Unemployed		
**Profession**	Scientific activities		
	Intermediate level		
	Administrative staff		
	Personal service		
	Machine operators		
	Unskilled workers		
**Professional practice**	Indoor		
	Outdoor		
**Monthly Family Income** (MW:760€)	Less than 1 MW		
	1-2 MW		
	3-4 MW		
	Doesn't know		
**Health Insurance**	Yes		
	No		

Table S2. Baseline characteristics of the cohort based on the questionnaire parameters (Continued)

		Number	Percentage (%)
Maternal characteristics (n=30)			
**Pre-pregnancy BMI** (kg/m^2)	Underweight (<18.5)		
	Normal weight (18.5-24.9)		
	Overweight (25-29.9)		
	Obesity (≥30)		
**Season of conception**	November to April		
	May to October		
**Mode of conception**	Natural/spontaneous		
	In vitro fertilization		
**Prenatal surveillance**	One appointment		
	Two or more		
**Number of pregnancies**	First pregnancy		
	2nd pregnancy		
	3rd or more		
**Parity**	Nulliparous		
	1 birth		
	2 births		
	3 or more		
**Previous History of Abortion**	Yes		
	No		
**Previous history of prematurity**	Yes		
	No		
**Smoking habits**	Never smoked		
	Stopped before pregnancy		
	Smoked in early pregnancy		
	Current smoker		
**Passive Smoking**	Yes		
	No		
**Alcohol habits**	Never drank		
	Stopped before pregnancy		
	Drank early in pregnancy		
	Currently drinks		
**Sleep Habits**	Less than 6 hours		
	6-7 hours		
	8-9 hours		
	More than 9 hours		
**Type of diet**	Mediterranean		
	Paleo/Paleolithic		
	Vegetarian/Vegan		
	Keto or Ketogenic		
	Macrobiotic Diet		
	Other		

Table S2. Baseline characteristics of the cohort based on the questionnaire parameters (Continued)

		Number	Percentage (%)
Maternal characteristics (n=30)			
**Consumption of vitamin D-rich foods per week**			
**Eggs**	Never		
	1-2 times		
	3-5 times		
	Every day		
**Oily fish**	Never		
	1-2 times		
	3-5 times		
	Every day		
**Milk and dairy products**	Never		
	1-2 times		
	3-5 times		
	Every day		
**Mushrooms**	Never		
	1-2 times		
	3-5 times		
	Every day		
**Liver steak**	Never		
	1-2 times		
	3-5 times		
	Every day		
**Chicken liver**	Never		
	1-2 times		
	3-5 times		
	Every day		
**Cod liver oil**	Never		
	1-2 times		
	3-5 times		
	Every day		
**Fish oil**	Never		
	1-2 times		
	3-5 times		
	Every day		
**Physical Activity**	Yes		
	No		
**Outdoor activity**	Yes		
	No		
**Weekly frequency of physical activity**	Less than 3 days		
	3-4 days		
	5 or more days		
**Daily duration of outdoor activity**	Less than 30 minutes		
	30 minutes to 1 hour		

Table S2. Baseline characteristics of the cohort based on the questionnaire parameters (Continued)

		Number	Percentage (%)
Maternal characteristics (n=30)			
**Time of outdoor activity**	Before 10 a.m.		
	Between 10 a.m. and 3 p.m.		
	After 3 p.m.		
**Sun exposure during outdoor activities**	Face and hands		
	Face, arms and hands		
	Face, arms, hands and legs		
**Use sunscreen**	Always		
	Only in summer		
	Before going to the beach		
	Before any outdoor activity		
	Never		
**Skin Type**	Type I		
(Fitzpatrick phototyping scale)	Type II		
	Type III		
	Type IV		
	Type V		
	Type VI		
**Pre-gestational pathology**	Yes		
	No		
**Gestational supplement with vitamin D**	Yes		
	No		
**Knowledge of the dosage of vitamin D**	Yes		
**in the supplement**	No		

Table S3. Baseline characteristics of the cohort based on clinical record parameters

		Number	Percentage (%)
**Season at the time of blood collection**	Spring		
	Summer		
	Fall		
	Winter		
**Perinatal period timing at the time of collection**	1st trimester		
	2nd trimester		
	3rd trimester		
	Postpartum		
**Daily dose of vitamin D supplementation**	Less than 400 UI		
	Between 400 and 600 UI		
	More than 600 up to 1000 UI		
	More than 1000 up to 2000 UI		
	Over 2000		
UI			
**Maternal vitamin D levels in the perinatal period**	Deficiency (<20 ng/mL)		
	Insufficiency (20-29.9 ng/mL)		
(n=30)	Sufficiency (≥ 30 ng/mL)		
**Maternal vitamin D levels in the prenatal period**	Deficiency (<20 ng/mL)		
	Insufficiency (20-29.9 ng/mL)		
(n=16)	Sufficiency (≥ 30 ng/mL)		
**Maternal vitamin D levels in the postnatal period**	Deficiency (<20 ng/mL)		
	Insufficiency (20-29.9 ng/mL)		
(n=15)	Sufficiency (≥ 30 ng/mL)		
**Collection for inflammatory biomarkers**	Yes		
	No		
**Gestational pathology**	Yes		
	No		
**Use of medication during pregnancy**	Yes		
	No		
**Gestational diabetes**	Yes		
	No		
**Gestational hypertension**	Yes		
	No		
**Gestational weight gain**	Excessive (>16 kg)		
	Adequate (11-16 kg)		
	Insufficient (<11Kg)		
**Maternal age at birth**	<20		
(years)	20-34		
	35-45		
	≥46		
**Type of delivery**	Eutocic		
	Dystocic		
**Dystocia by caesarean section**	Yes		
	No		
**Reason for caesarean section**	Elective		

Emergency

Table S3. Baseline characteristics of the cohort based on clinical record parameters (Continued)

		**Number**	**Percentage (%)**
**Cause of caesarean section emergency**	Maternal cause		
	Fetal cause		
**Preterm birth**	Yes		
	No		
**Preterm birth**	Elective		
	Spontaneous		
**Adverse clinical outcomes**	No adverse effects		
	One adverse effect		
	Two adverse effects		
Neonatal outcomes			
**Gestational age at birth**	Preterm (<37 weeks)		
	Term (≥37 weeks)		
**Season at birth**	November to April		
	May to October		
**Sex**	Male		
	Female		
**Apgar score 1st minute**	< 7		
	≥7		
**Apgar score 5th minute**	<7		
	≥7		
**Need for resuscitation maneuvers**	Yes		
	No		
**Birth weight** (gr)	LBW (1500-2499)		
	Underweight (2500-2999)		
	Normal weight (3000-3999)		
**Length at birth** (cm)	Short birth length (<50)		
	Normal (≥50)		
**Head circumference** (cm)	Small head circumference (<35)		
	Normal (≥35)		
**Presence of meconium**	Yes		
	No		
**Duration of neonatal hospitalization**	Normal (2-3 days)		
	Above normal (>3 days)		
**Admission to the NICU**	Yes		
	No		
**Neonatal adverse clinical effects**	No adverse effects		
	One adverse effect		
	Two adverse effects		
